# Effects of intensified conditioning on Epstein-Barr virus and cytomegalovirus infections in allogeneic hematopoietic stem cell transplantation for hematological malignancies

**DOI:** 10.1186/1756-8722-5-46

**Published:** 2012-08-02

**Authors:** Li Xuan, Fen Huang, Zhiping Fan, Hongsheng Zhou, Xian Zhang, Guopan Yu, Yu Zhang, Can Liu, Jing Sun, Qifa Liu

**Affiliations:** 1 Department of Hematology, Nanfang Hospital, Southern Medical University, Guangzhou, 510515, China

**Keywords:** Epstein-Barr virus, Cytomegalovirus, Conditioning, Hematological malignancies, Allogeneic hematopoietic stem cell transplantation

## Abstract

**Background:**

Intensified conditioning regimens (increasing the intensity of standard myeloablative conditioning) for hematological malignancies in allogeneic hematopoietic stem cell transplantation (allo-HSCT) could reduce the relapse rate of the underlying disease, but it might simultaneously increase the transplant-related mortality including the mortality of infections. To explore whether intensified conditioning affected Epstein-Barr virus (EBV) and cytomegalovirus (CMV) infections, 185 patients undergoing allo-HSCT were enrolled.

**Methods:**

A total of 104 cases received standard and 81 intensified conditioning. Cyclosporine A (CsA) withdrawal and/or donor lymphocyte infusion (DLI) were conducted in high-risk patients. The EBV-DNA and CMV-DNA levels of blood were monitored regularly by quantitative real-time polymerase chain reaction (RQ-PCR) and immune reconstitution of recipients were analyzed by flow cytometry.

**Results:**

The 3-year cumulative incidence of EBV viremia, EBV-associated diseases and mortality of EBV-associated diseases were 25.3% ± 4.6%, 10.5% ± 3.4% and 0.0% ± 0.0% in the standard group, compared with 45.6% ± 6.5%, 26.0% ±5.3% and 7.3% ± 3.1% in the intensified group (*P* = 0.002, *P* = 0.002, *P* = 0.008). The 3-year cumulative incidence of CMV viremia and CMV-associated diseases, mortality of CMV-associated diseases and incidence of bacterial and fungal infections were similar between the two groups (*P* = 0.855, *P* = 0.581, *P* = 0.933, *P* = 0.142, *P* = 0.182, respectively). Multivariate analysis showed that intensified conditioning was one of the risk factors for EBV viremia and EBV-associated diseases (*P* = 0.037, *P* = 0.037), but it had no effects on CMV infections. The percentage of CD4^+^ T cells and CD4^+^/CD8^+^ ratio at 3 months post-transplantation were lower in the intensified group (*P* = 0.032, *P* = 0.022). The 3-year OS and DFS in the standard group were 62.2% ± 5.8% and 60.6% ± 5.6%, compared with 51.6% ± 6.2% and 51.1% ± 5.9% in the intensified group (*P* = 0.029, *P* = 0.063).

**Conclusions:**

Intensified conditioning represents a promising approach for high-risk hematological malignancies, although it affects early immune reconstitution of recipients and increases the incidence and mortality of EBV infections.

## Introduction

Allogeneic hematopoietic stem cell transplantation (allo-HSCT) is a curative approach for hematological malignancies [1,2]. Besides graft-versus-host disease (GVHD), the two main causes of death after allo-HSCT remain relapse of malignancy and infections [3,4]. The relapse rate exceeds 50% in patients with refractory hematological malignancies with a standard myeloablative (MA) regimen consisting of total body irradiation (TBI)/busulfan (Bu) combined with cyclophosphamide (CY) [5]. Reduced-intensity conditioning (RIC) regimens have been advocated to reduce transplantation-associated toxicity in elderly or medically unfit patients [6,7]; however, disappointing results have been reported with RIC transplantation in patients with refractory hematological malignancies [8,9]. Some studies suggested that intensified conditioning regimens, which increased the intensity of standard myeloablative conditioning, could reduce tumor relapse, but it might simutaneously increase the transplant-related mortality (TRM) including the mortality of infections [10-12]. Therefore, overall survival (OS) did not improve significantly. To improve the outcomes of allo-HSCT for refractory hematological malignancies, we introduced a strategy of sequential intensified conditioning and early rapid tapering of prophylactic immunosupressants therapy for GVHD during the early stage after transplantation [13]. The results of this trial suggested that this strategy might reduce tumor relapse. Moreover, the nonrandomized data suggested that this strategy did not increase the incidence and mortality of bacterial and fungal infections [13], but the effect of this strategy on opportunistic viral infections needed further investigation.

Opportunistic viral infections, especially Epstein Barr virus (EBV) and cytomegalovirus (CMV) infections are one of the common complications after allo-HSCT. Both primary infections and reactivations of EBV and CMV may result in life-threatening diseases in recipients of allo-HSCT [14-17]. The occurrence of EBV and CMV infections and reactivations is influenced by several factors and closely related to the immune function [18-21]. Some studies showed that the intensity of conditioning might affect immune reconstitution of recipients after allo-HSCT [22,23]. To explore whether intensified conditioning affected EBV and CMV infections, we prospectively studied the incidence and mortality of EBV and CMV infections in the recipients of allo-HSCT following intensified or standard conditioning.

## Methods

### Patients

From February 2009 to December 2011, 189 consecutive patients with hematological malignancies received allo-HSCT in our single institution. A total of 185 cases were enrolled in this prospective study, and 4 cases who died from regimen-related toxicity (RRT) or bacterial infection before hematopoietic reconstitution were not included. The median age was 28.0 years (range 12–63 years). Seventy-two patients were female, and 113 were male. The primary diseases included acute leukemia (AL, n = 149), chronic myeloid leukemia (CML, n = 28), lymphoma (n = 5), myelodysplastic syndrome (MDS, n = 2) and blastic plasmacytoid dendritic cell neoplasm (n = 1). One hundred and twenty-five patients were in the status of complete remission (CR) (including patients with CML-chronic phase [CP]), and 60 were not in CR (NR) at the time of transplantation. All recipients were EBV-DNA negative in blood and 99 were EBV-VCA (viral capsid antigen, IgG) seropositive before transplantation. Two donors were EBV-DNA positive and became EBV-DNA negative with antiviral agents before collection of stem cells; 111 donors were EBV-seropositive. Seven recipients and nine donors were CMV-DNA positive in blood before transplantation. After antiviral treatment, they were all CMV-DNA negative at the time of transplantation. One hundred and sixty-nine recipients and 171 donors were CMV-IgG positive (Table 1). The study was performed in accordance with the modified Helsinki Declaration, and the protocol was approved by our ethical review boards before study initiation. All recipients, donors and/or guardians provided written informed consent.

**Table 1:** Patient, donor and transplants characteristics

**Patient characteristics**	**Intensified myeloablative conditioning (n = 81)**	**Standard myeloablative conditioning (n = 104)**	**P-value**
Female/Male	31(38.3%)/50(61.7%)	41(39.4%)/63(60.6%)	NS
Median age, years (range)	26(14–54)	28(12–63)	NS
Disease			
ALL	42 (51.85%)	23 (22.1%)	P<0.001
AML	17 (21.0%)	43(41.4%)	
ALAL	12 (14.8%)	12 (11.5%)	
CML	6 (7.4%)	22 (21.2%)	
HD/NHL	3(3.7%)	2 (1.9%)	
MDS	0 (0%)	2 (1.9%)	
BPDCN	1 (1.25%)	0 (0%)	
Disease status at the time of transplants			
CR (including CML-CP)	31 (38.3%)	94 (90.4%)	P<0.001
NR (including CML-AP and BC)	50 (61.7%)	10 (9.6%)	
EBV serostatus			
D-/R-	15(18.5 %)	21(20.2 %)	NS
D+/R-	23(28.4%)	27(26.0%)	
D-/R+	16(19.8%)	22(21.1%)	
D+/R+	27(33.3%)	34(32.7%)	
CMV serostatus			
D-/R-	4(4.9%)	5(4.8%)	NS
D+/R-	3(3.7%)	4(3.85%)	
D-/R+	3(3.7%)	2(1.95%)	
D+/R+	71(87.7%)	93(89.4%)	
Donor type			
Sibling donor	44(54.3%)	73 (70.2%)	P = 0.010
Family donor	11 (13.6%)	3 (2.9%)	
Unrelated donor	26(32.1%)	28 (26.9%)	
HLA typing			
HLA-identical	49 (60.5%)	79 (76.0%)	NS
One allele mismatched	15 (18.5%)	15 (14.4%)	
Two alleles mismatched	8 (9.9%)	8 (7.7%)	
Three alleles mismatched	1 (1.2%)	1 (0.95%)	
Four alleles mismatched	6 (7.4%)	1 (0.95%)	
Five alleles mismatched	2 (2.5%)	0 (0.0%)	
Stem cell source			
PBSCs	63 (77.8%)	90 (86.5%)	NS
BM	1 (1.2%)	0 (0.0%)	
PBSCs + BM	17(21.0%)	14 (13.5%)	
Median CD34^+^ cells per graft, ×10^6^/kg (range)	8.8(5.4-11.9)	8.1(5.2-11.7)	NS
Conditioning			
TBI + CY		36 (34.6%)	NA
Bu + CY		40 (38.5%)	
Bu + Flu		28(26.9%)	
Flu + Ara-c + TBI + CY	49 (60.5%)		
TBI + CY + VP-16	32 (39.5%)		
GVHD prophylaxis			
CsA	4 (5.0%)	2 (1.9%)	NS
CsA + MTX	33 (40.75%)	62 (59.6%)	
CsA + MTX + ATG	28 (34. 5%)	27 (26.0%)	
CsA + MTX + MMF	3 (3.7%)	2 (1.9%)	
CsA + MTX + ATG + MMF	13 (16.05%)	11 (10.6%)	

*ALL* = acute lymphoblastic leukemia; *AML* = acute myelogenous leukemia; *ALAL* = acute leukemia of ambiguous lineage; *CML* = chronic myelogenous leukemia; *HD* = Hodgkin’s disease; *NHL* = Non-Hodgkin’s lymphoma; *MDS* = myelodysplastic syndromes; *BPDCN* = blastic plasmacytoid dendritic cell neoplasm;*CR* = complete remission; *CP* = chronic phase; *NR* = not in CR; *AP* = accelerated phase; *BC* = blastic crisis; *D* = donor; *R* = recipient; *PBSCs* = peripheral blood stem cells; *BM* = bone marrow; *TBI* = total body irradiation; *CY* = cyclophosphamide; *BU* = busulfan; *FLU* = fludarabine; *Ara-c* = cytarabine; *VP16* = etoposide; *GVHD* = graft-versus-host disease; *CSA* = cyclosporine A; *MTX* = methotrexate; *ATG* = antithymocyte globulin; *MMF* = mycophenolate mofetil; *NS* = not significant; *NA* = not applicable.

### Conditioning regimens

Five conditioning regimens, including three standard MA and two intensified MA conditioning regimens were administrated. The standard conditioning was as follows: ① TBI (4.5 Gy/day, -5, -4 days) + CY (60 mg/kg/day, -3, -2 days) in 36 recipients; ② Bu (3.2 mg/kg/day, -7 to −4 days) + CY (60 mg/kg/day, -3, -2 days) in 40 recipients; ③ Bu (3.2 mg/kg/day, -6 to −3 days) + Flu (fludarabine, 30 mg/m^2^, -6 to −2 days) in 28 recipients. The intensified conditioning included the following: ① TBI (4.5 Gy/day, -5, -4 days) + CY + VP-16 (etoposide, 10–15 mg/kg/day, -3, -2 days) in 32 recipients; ② Flu (30 mg/m^2^/day, -10 to −6 days) + Ara-C (cytarabine, 2.0 g/m^2^/day, -10 to −6 days) plus TBI (4.5 Gy/day, -5, -4 days) + CY in 49 recipients [13]. The alternative rules of conditioning depended on the high risk factors for primary diseases and comorbidities at the time of transplantation. Generally, except those with severe comorbidities, patients with high-risk genetics and/or in NR at the time of transplantation all received intensified conditioning; patients with intermediate/low-risk genetics and in CR at the time of transplantation all received standard conditioning (Table 1).

### Cyclosporine A (CsA) withdrawal and donor lymphocyte infusion (DLI) in high-risk patients

According to the following criteria, CsA withdrawal and/or DLI were conducted in all patients with acute lymphoblastic leukemia (ALL) and high-risk factors (high-risk genetics or NR/>CR2 [second complete remission] at the time of transplantation). Depending on whether donor lymphocytes were available, CsA was withdrawn in two ways in patients who did not experience acute GVHD (aGVHD) by day +30 post-transplantation: if donor lymphocytes were unavailable, CsA was withdrawn rapidly in a stepwise fashion (ie, total dose reduced by 20%/week); if they were available, CsA was withdrawn in a stepwise fashion (ie, total dose reduced by 10%/week) and G-CSF mobilized donor lymphocytes (1.0 × 10^8^/kg, once a month, 4 doses totally) would be infused in patients without II° or more than II° aGVHD by day + 60 post-transplantation. Once patients developed GVHD after DLI, DLI would stop and methylprednisolone was added to the regimen.

### Prophylaxis and treatment for GVHD

CsA alone or CsA plus MTX (methotrexate) (on days +1 and +3) were administered in patients with NR undergoing HLA-matched sibling donor transplantation, and CsA plus MTX (on days +1, +3 and +6) were administered in patients with CR undergoing HLA matched sibling donor transplants for GVHD prophylaxis. CsA + MTX + ATG (antithymocyte globulin, for total doses of 6–10 mg/kg, on days −3 to −1 or −4 to 0) and/or MMF (mycophenolate) were used in patients undergoing HLA-mismatched related and unrelated donor transplants. Methylprednisolone (1–2 mg/kg/day) was used to treat aGVHD. ATG or ATG combined with CD25 monoclonal antibody and other immunodepressants were used to treat glucocorticosteroid-resistant aGVHD. Corticosteroids and CsA were used initially to treat chronic GVHD (cGVHD) and were used in combination with various immunosuppressive agents to treat cGVHD that was unresponsive to initial therapy.

### Infection prophylaxis

Oral sulfamethoxazole and norfloxacin were given to all patients. Acyclovir was given daily from the beginning of conditioning therapy to engraftment, and it was then administered daily for 7 days every 2 weeks until 1 year after transplantation. Ganciclovir was given for 2 weeks before transplantation for prophylaxis of CMV infections, and was administered once again when CMV viremia occurred. Antifungal agents were administered 5 days before transplantation. Fluconazole (0.3 g/day) or itraconazole (0.4 g/kg.d) was used for up to +60 days post-transplantation in patients with no history of invasive fungal infection (IFI); those with a history of IFI received itraconazole (0.4 g/day), voriconazole (0.4 g/day), caspofungin (50 mg/day) or Am-Bisome (2 mg/kg.day) intravenously. Oral itraconazole or voriconazole was started when the peripheral white blood cell count exceeded 2.0 × 10^9^/L and was discontinued after 90 days post-transplantation.

### Monitoring of EBV-DNA and CMV-DNA levels in blood

Generally, the EBV-DNA and CMV-DNA levels of blood were monitored weekly for three months after transplantation. During the 4th to 9th month post-transplantation, the monitoring frequency was once every two weeks; the 10th to the 24th month, once a month; the 25th to 36th month, once every three months. If EBV-DNA or CMV-DNA was positive, it was monitored twice a week.

The DNA levels of EBV and CMV in blood were detected by quantitative real-time polymerase chain reaction (RQ-PCR) [24,25]. The plasma (50 μl) was mixed with 50 μl of nucleic acid extract, and the mixture was heated at 99°C for 10 minutes and then centrifuged at 13000 rpm for 10 minutes. The supernatant was collected for the next step. The PCR conditions for EBV were as follows: 37°C for 2 mins and 94°C for 2 mins followed by 40 cycles at 93°C for 15 s and 60°C for 1 min. The sequences of the TaqMan probes and primers for EBV were as follows: EBV TaqMan probe.

(FAM)-TCTGCTGTTGTTTCTGTCTCACCTACCGG-(TAMRA); EBV forward primer, 5’-CCAGTGCTGTGATCGAGCATCT-3’; and EBV reverse primer, 5’-CTGCTGACAAACTGCTGCATTC-3’. For CMV, the PCR conditions were as follows: 1 cycle at 50°C for 2 mins, 95°C for 10 mins and 45 cycles at 95°C for 15 s and 60°C for 1 min. The forward primer was 5’-GAAGGTGCAGGTGCCCTG-3’, the reverse primer 5’-GTGTCGACGAACGACGTACG-3’ and the probe (FAM)-ACGGTGCTGTAGACCCGCATACAAA-(TAMRA). The normal threshold for EBV-DNA and CMV-DNA copies in plasma provided by the manufacturer (ZJ Bio-Tech Co.,Ltd., Shanghai, China) was less than 500 copies/ml. EBV-DNA or CMV-DNA was considered positive when the copies exceeded 500 copies/ml.

### Intervention for EBV and CMV viremia

Once EBV-DNA or CMV-DNA in the blood was positive, the viral loads would be detected once again the next day. When EBV-DNA in the blood was positive twice consecutively, several measures of control were taken, including administration of antiviral agents (ganciclovir, acyclovir or foscarnet), immunoglobulin (0.4 g/kg/d × 3d) and reduction of immunosuppression if the condition of the patient was acceptable. If EBV-DNA in the blood was continuously positive four times with a rising trend, anti-CD20 antibody (rituximab, 375 mg/m^2^) was administered weekly until EBV-DNA was negative or for a total of 4 weeks.

When CMV-DNA in the blood was positive twice consecutively, ganciclovir or foscarnet was administrated. If CMV-DNA in the blood was continuously positive four times with a rising trend, several measures of control were taken, including immunoglobulin (0.4 g/kg/d × 3d), reduction of immunosuppression and the combination of antiviral agents (ganciclovir and foscarnet).

### Diagnosis of EBV-and CMV-associated diseases

EBV-associated diseases were classified into EBV-associated post-transplant lymphoproliferative diseases (PTLD) and EBV-associated other diseases. The diagnosis of EBV-associated PTLD was according to the criteria of World Health Organization (WHO) [26,27]. The diagnosis of EBV-associated other diseases was based on the criteria of the European Conference on Infections in Leukemia and literatures [17,28,29], which included EBV-associated fever without tissue involvement, EBV-associated diseases with tissue other than lymphatic tissue involvement.

CMV-associated diseases were defined according to published recommendations [15]. Briefly, CMV-associated disease was defined by the presence of clinical symptoms or signs of end organ disease, combined with the evidence of CMV infection in a tissue biopsy specimen. CMV pneumonia was diagnosed on the basis of signs and symptoms compatible with a diagnosis of pneumonia (hypoxemia, x-ray) and a bronchoalveolar lavage (BAL) fluid or lung biopsy specimen positive for CMV by immunohistology. CMV gastrointestinal (GI) disease was diagnosed when GI signs or symptoms occurred, and evidence of CMV in the GI tract was diagnosed by immunohistochemistry or in situ hybridization from biopsy specimens. CMV encephalitis was defined by the identification of central nervous system symptoms together with the detection of CMV-DNA in cerebrospinal fluid samples.

Once EBV- or CMV-associated diseases were considered or diagnosed, other viruses DNA including herpes simplex virus (HSV) -types 1 and 2, adenovirus (ADV), varicella zoster virus (VZV), human herpesvirus 6–8 (HHV6-8), parvovirus B19 and BK virus (BKV) were detected in blood (VZV and parvovirus B19 were detected by kit provided by ZJ Bio-Tech Co.,Ltd., Shanghai. HSV, ADV, HHV6-8 and BKV were detected by kit provided by Huayin medical technology Co.,Ltd., Guangzhou.). BKV were measured using RQ-PCR (Eppendorf AG, Hamburg, Germany), and other viruses were measured using qualitative PCR.

### Treatment of EBV- and CMV-associated diseases

Once EBV-associated diseases were diagnosed, several measures would be taken promptly, including antiviral agents, reduction of immunosuppression, rituximab, combination chemotherapy, DLI and EBV-specific cytotoxic lymphocyte (EBV-CTL) treatment.

Once CMV-associated diseases were diagnosed, several measures would also be taken promptly, including administration of ganciclovir and foscarnet, immunoglobulin (0.4 g/kg/d × 3d) and reduction of immunosuppression.

### Flow cytometry analysis

T lymphocyte subgroups (CD3^+^, CD3^+^CD4^+^, CD3^+^CD8^+^), B lymphocytes (CD19^+^) and NK cells (CD16^+^ CD56^+^) in peripheral blood of recipients were analyzed by flow cytometry, respectively, at 1, 3 and 6 months after transplantation. FITC-conjugated mouse anti-human CD3 (HIT3a), APC-conjugated anti-human CD4 (RPA-T4), PE-conjugated anti-human CD8 (HIT8a), PerCP-Cy5.5-conjugated anti-human CD19 (HIB19), PE-conjugated anti-human CD16 (3 G8), PerCP-Cy5.5-conjugated anti-human CD56 (B159) were purchased from BD Pharmingen. Freshly isolated peripheral blood were incubated with FITC-, PE-, APC- and PerCP-Cy5.5-conjugated mAbs or their isotype control Abs for 30 min at 4°C, followed by hemolysis liquid washing and phosphate-buffered saline solution washing. All samples were assayed by BD FACSCanto^TM^ II (BD Biosciences) and the acquired data were further analyzed using BD-FACSDiva Software. Flow cytometric results were represented as percentage positive.

### Evaluation points and statistics

Our data was analyzed on May 31, 2012. The main evaluation points included EBV and CMV infections within 3 years post-transplantation as well as bacterial and fungal infections within 100 days post-transplantation. The secondary evaluation points included hematopoietic engraftment, primary disease response, aGVHD, cGVHD, immune reconstitution, recurrence and survival. Comparisons of categorical variables were made by means of chi-squared and Fisher exact tests for small numbers. Differences between numerical variables were calculated by means of the Mann–Whitney *U*-test. Incidence of time-dependent variables was estimated by the method of Kaplan-Meier. Intervals were measured from the day of transplantation until first diagnosis of EBV or CMV infections or until the last day of follow-up, transplant-related death or relapse. Univariate and multivariate Cox regression models were used to analyze risk factors for EBV and CMV infections after transplantation as well as OS and DFS (disease-free survival). EBV and CMV infections as well as OS and DFS were entered as time-dependent covariates. Variables for the multivariate models were selected with backward stepwise elimination with significance exceeding 0.05 as the criterion for removal from the models. A variable indicating whether patients were in the intensified or standard group was included in the models regardless of its significance.

## Results

### Patient, donor and transplants characteristics

The characteristics of patients, donors and transplants are summarized in Table 1. There were significant differences between standard and intensified group in the category of diseases (*P*<0.001), disease status at the time of transplantation (*P*<0.001) and donor type (*P* = 0.010). As could be seen from the comparison, more patients in the intensified group were cases with ALL and/or in NR, receiving more family or unrelated donor transplants compared with standard group.

### Hematopoietic engraftment and primary disease response

Of the 189 consecutive patients undergoing transplantation, 4 cases (3 in the intensified group, 1 in the standard group) died from RRT or bacterial infection before hematopoietic reconstitution and were not included. Regeneration of neutrophil counts > 0.5 × 10^9^/L took a median of 11 days (range 9–22 days) and 12 days (range 9–31 days) in the standard and intensified group(*P* = 0.486), respectively. Platelet counts > 20 × 10^9^/L were reached after a median of 12 days (range 9–40 days) and 13 days (range 9–70 days) in the standard and intensified group (*P* = 0.029), respectively. The sixty patients in NR at the time of transplantation, including 50 cases in the intensified group and 10 in the standard group, all achieved CR by day +30 post-transplantation.

### CsA withdrawal and DLI

CsA was withdrawn in 42 (40.4%) cases in the standard group and 50 (61.7%) in the intensified group according to the criteria aforementioned (*P* = 0.004). 16.3% (17/104) and 45.7% (37/81) cases received DLI in the standard and intensified group, respectively (*P*<0.001). Thirty-eight cases who met the criteria of DLI did not receive DLI because of the limitation of the donor lymphocytes source, including 25 cases in the standard group and 13 in the intensified group (*P* = 0.182).

### GVHD

Ninety-six cases developed aGVHD including 13 after DLI; 80 of 164 cases surviving more than 100 days developed cGVHD, including 20 after DLI. Grade I-IV aGVHD occurred in 49 of 104 (47.1%) patients in the standard group (grade I, n = 13; grade II, n = 29; grade III, n = 5; grade IV, n = 2), compared with 47 of 81 (58.0%) cases in the intensified group (grade I, n = 8; grade II, n = 28; grade III, n = 7; grade IV, n = 4) (*P* = 0.141). cGVHD occurred in 43 of 94 (45.7%) patients in the standard group (limited, n = 25; extensive, n = 18), compared with 37 of 70 (52.9%) cases in the intensified group (limited, n = 27; extensive, n = 10) (*P* = 0.367). In order to rule out the effects of immunosuppressants withdrawal and DLI on GVHD, the incidence of aGVHD I-IV and II- IV by day +30 was compared in the two groups. Acute GVHD I-IV and II- IV occurred in 31.7% (33/104) and 4.81% (5/104) patients by day +30 post-transplantation in the standard group, compared with 43.2% (35/81) and 12.3% (10/81) cases in the intensified group (*P* = 0.108, *P* = 0.062, respectively).

### Infections within 100 days post-transplantation

Within the first 100 days post-transplantation, 101 cases developed 150 episodes of infections. Comparing standard versus intensified conditioning, 20 vs 13 had bacterial infections, 2 vs 5 had IFI, 9 vs 7 had viral infections except CMV and EBV viremia, 15 vs 22 had mixed infections and 5 vs 3 had infections of unknown etiology. The infection rates and the incidence of bacterial and fungal infections within 100 days post-transplantation were 49.0%, 32.7% and 11.5% in the standard group, compared with 61.7%, 43.2% and 18.5% in the intensified group (*P* = 0.085, *P* = 0.142, *P* = 0.182, respectively). Eight cases died of infections within 100 days post-transplantation, including three who died of CMV-associated diseases and two who died of EBV-associated diseases.

### EBV viremia and EBV-associated diseases

With a median follow-up of 319 days post-transplantation (range, 27 to 1194 days), 57 cases (30.4%) developed EBV viremia and 28 (15.1%) developed EBV-associated diseases including 16 EBV- PTLD and 12 EBV-associated other diseases (7 EBV-associated fever, 1 encephalitis, 1 myelitis, 1 encephalitis with lung involvement, 1 encephalitis with lung and liver involvement and 1 pneumonia). EBV-associated diseases occurred in 2 (2%), 1 (50%) and 9 (34.6%) cases receiving transplants from matched sibling donor, matched family donor and matched unrelated donor; they occurred in 4 (23.5%), 4 (33.3%) and 8 (28.6%) cases from mismatched sibling donor, mismatched family donor and mismatched unrelated donor. The median time to onset of EBV viremia and EBV-associated diseases was 51 days (range, 22–368 days) and 63 days (range, 22–289 days) post-transplantation, respectively.

**Figure 1:**
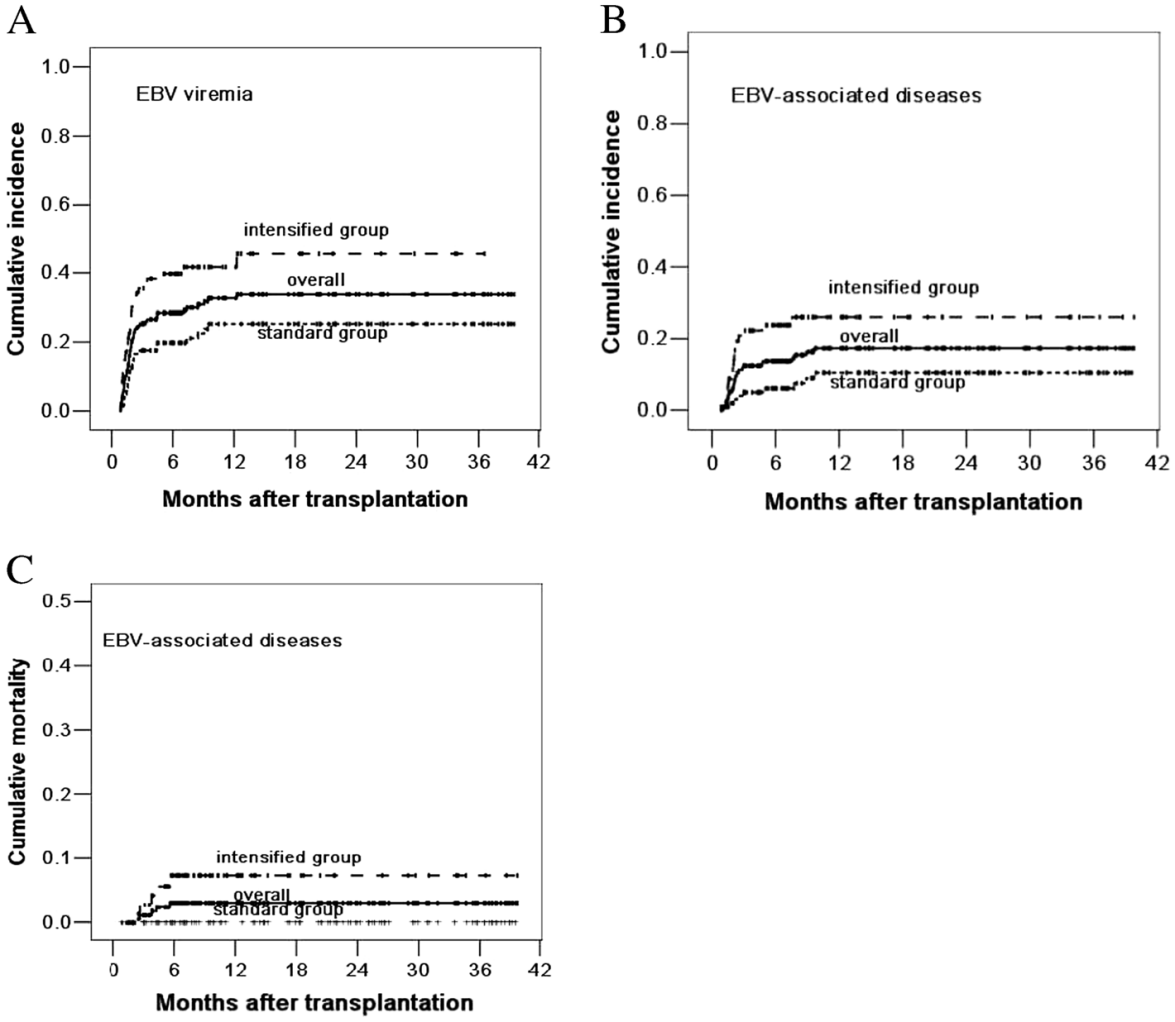
**Cumulative incidence of EBV viremia (A), EBV-associated diseases (B) and mortality of EBV-associated diseases (C).** The 3-year cumulative incidence of EBV viremia and EBV-associated diseases were 25.3% ±4.6% and 10.5% ±3.4% in the standard group, compared with 45.6% ± 6.5% and 26.0% ±5.3% in the intensified group (*P* = 0.002, *P* = 0.002). The 3-year cumulative mortality of EBV-associated diseases was 0.0% ± 0.0% and 7.3% ± 3.1% in the standard and intensified group, respectively (*P* = 0.008)

EBV viremia occurred in 24 (23.1%) cases in the standard group and 33 (40.7%) cases in the intensified group (*P* = 0.010). EBV-associated diseases occurred in 9 (8.7%) cases in the standard group, including 4 EBV-associated fever, 4 PTLD and 1 encephalitis with lung and liver involvement; they occurred in 19 (23.5%) cases in the intensified group, including 3 EBV- associated fever, 12 PTLD, 1 encephalitis, 1 myelitis, 1 encephalitis with lung involvement and 1 pneumonia (*P* = 0.005). The 3-year cumulative incidence of EBV viremia and EBV-associated diseases were 25.3% ±4.6% and 10.5% ±3.4% in the standard group, compared with 45.6% ± 6.5% and 26.0% ±5.3% in the intensified group (*P* = 0.002, *P* = 0.002, respectively, log-rank test, Figure 1A and 1B). No case in the standard group and five cases in the intensified group died of EBV-associated diseases. The 3-year cumulative mortality of EBV-associated diseases was 0.0% ± 0.0% and 7.3% ± 3.1% in the standard and intensified group, respectively (*P* = 0.008) (Figure 1C).

### CMV viremia and CMV-associated diseases

Seventy-seven cases (41.6%) developed CMV viremia and 10 (4.9%) developed CMV-associated diseases including 4 CMV pneumonia, 1 CMV encephalitis, 3 CMV enteritis, 1 CMV encephalitis together with enteritis as well as 1 CMV pneumonia together with encephalitis. The median time to onset of CMV viremia and CMV-associated diseases was 41 days (range, 11–410 days) and 106 days (range, 45–198 days) post-transplantation, respectively.

**Figure 2:**
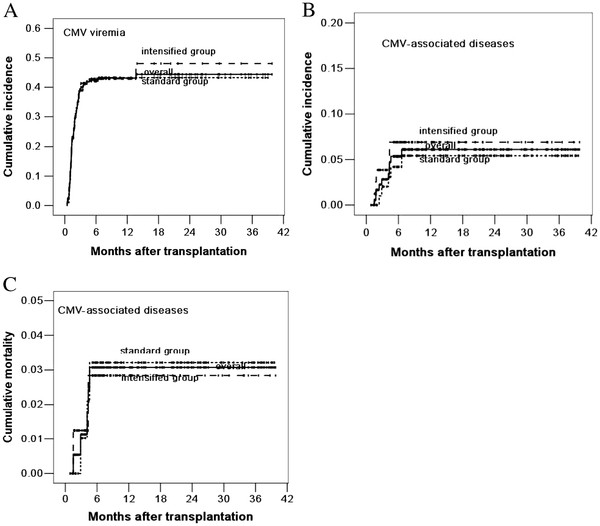
**Cumulative incidence of CMV viremia (A), CMV-associated diseases (B) and mortality of CMV-associated diseases (C).** The 3-year cumulative incidence of CMV viremia and CMV-associated diseases were 43.3% ± 4.9% and 5.4% ± 2.4% in the standard group, compared with 48.1% ± 7.1% and 6.9% ± 3.0% in the intensified group (*P* = 0.855, *P* = 0.581). The 3-year cumulative mortality of CMV-associated diseases was 3.2% ± 1.8% and 2.9% ± 2.0% in the standard and intensified group, respectively (*P* = 0.933)

CMV viremia occurred in 43 (41.3%) cases in the standard group and 34 (42.0%) cases in the intensified group (*P* = 0.931). CMV-associated diseases occurred in 5 (4.8%) cases in the standard group and 5 (6.2%) cases in the intensified group (*P* = 0.684). The 3-year cumulative incidence of CMV viremia and CMV-associated diseases were 43.3% ± 4.9% and 5.4% ± 2.4% in the standard group, compared with 48.1% ± 7.1% and 6.9% ± 3.0% in the intensified group (*P* = 0.855, *P* = 0.581, respectively, log-rank test, Figure 2A and 2B). Three cases in the standard group and two cases in the intensified group died of CMV-associated diseases. The 3-year cumulative mortality of CMV-associated diseases was 3.2% ± 1.8% and 2.9% ± 2.0% in the standard and intensified group, respectively (*P* = 0.933) (Figure 2C).

### Risk factors for EBV and CMV infections

Univariate and multivariate analysis about the risk factors for EBV and CMV infections are showed in Table 2. On multivariate analysis, only use of ATG (relative risk [*RR*] =15.554, 95% confidence interval [*CI*]: 6.598-36.664, *P*<0.001; *RR* =19.216, 95% *CI*: 4.525-81.598, *P*<0.001, respectively) and intensified conditioning regimens (*RR* =1.759, 95% *CI*: 1.034-2.991, *P* = 0.037; *RR* =2.290, 95% *CI*: 1.051-4.994, *P* = 0.037, respectively) were the risk factors for EBV viremia and EBV-associated diseases; HLA mismatch (*RR* =2.441, 95% *CI*: 1.551-3.840, *P*<0.001) and early CsA withdrawal (*RR* =2.112, 95% *CI*: 1.307-3.412, *P* = 0.002) were the risk factors for CMV viremia; aGVHDII-IV(*RR* =12.554, 95% *CI*: 1.570-100.401, *P* = 0.017) was the only risk factor for CMV-associated diseases. In contrast, age, sex, EBV and CMV serological status, use of Flu and the occurrence of cGVHD did not show any significant influence on the risk of EBV and CMV infections.

**Table 2:** Univariate and multivariate analyses of risk factors for EBV and CMV infections after allo-HSCT

**Risk factors**	**EBV viremia**		**EBV-associated diseases**		**CMV viremia**		**CMV-associated diseases**	
	**Univariate**	**Multivariate (RR)**	**Univariate**	**Multivariate (RR)**	**Univariate**	**Multivariate (RR)**	**Univariate**	**Multivariate (RR)**
male vs female	NS	NS	NS	NS	NS	NS	NS	NS
Age, <20 years vs ≥20 to ≤ 40 years vs > 40 years	NS	NS	NS	NS	NS	NS	NS	NS
Disease status, CR vs NR	P = 0.029	NS	NS	NS	NS	NS	NS	NS
Related vs unrelated donor	P<0.001	NS	P<0.001	NS	NS	NS	NS	NS
HLA typing, matched vs mismatched	P<0.001	NS	P<0.001	NS	P<0.001	P<0.001 (2.441)	NS	NS
Standard vs intensified conditioning	P = 0.002	P = 0.037 (1.759)	P = 0.006	P = 0.037 (2.290)	NS	NS	NS	NS
EBV serological matches vs mismatches	NS	NS	NS	NS	NS	NS	NS	NS
CMV serological matches vs mismatches	NS	NS	NS	NS	NS	NS	NS	NS
ATG vs no ATG	P<0.001	P<0.001 (15.554)	P<0.001	P<0.001 (19.216)	P<0.001	NS	P = 0.006	NS
Flu vs no Flu	NS	NS	NS	NS	NS	NS	NS	NS
Acute GVHD II-IV vs 0-I	NS	NS	P = 0.032	NS	P<0.001	NS	P = 0.002	P = 0.017 (12.554)
Chronic GVHD vs no cGVHD	NS	NS	NS	NS	NS	NS	NS	NS
Early CsA withdrawal vs no early withdrawal	NS	NS	NS	NS	P<0.001	P = 0.002 (2.112)	NS	NS
DLI vs no DLI	NS	NS	NS	NS	P = 0.010	NS	NS	NS

*RR* = relative risk; *vs* = versus; *CR* = complete remission; *NR* = not in CR; *HLA* = human leukocyte antigen; *EBV* = Epstein-Barr virus; *CMV* = Cytomegalovirus; *ATG* = anti-thymocyte globulin; *FLU* = fludarabine; *GVHD* = graft-versus-host disease; *CSA* = cyclosporine A; *DLI* = donor lymphocyte infusion; *NS* = not significant.

### Immune reconstitution

The total lymphocyte counts at 1, 3 and 6 months after transplantation were (0.686 ± 0.453) × 10^9^/L, (1.463 ± 0.878) × 10^9^/L and (1.512 ± 0.916) × 10^9^/L in the standard group, compared with (0.709 ± 0.390) × 10^9^/L, (1.237 ± 0.682) × 10^9^/L and (1.566 ± 0.763) × 10^9^/L in the intensified group (*P* = 0.510, *P* = 0.082, *P* = 0.354, respectively). The percentage of CD4^+^ T cells and ratio of CD4^+^/CD8^+^ T cells in the standard group at 3 months post-transplantation were significantly higher than that in the intensified group (*P* =0.032, *P* =0.022). The percentages of CD3^+^ T cells, CD8^+^ T cells, CD19^+^ B cells and CD16^+^ CD56^+^ NK cells at 3 months post-transplantation were similar between standard and intensified group (all *P*>0.05). The percentages of all cell subsets at 1 and 6 months post-transplantation did not differ significantly between standard and intensified group (all *P*>0.05).

### OS and DFS

With a median follow up of 10.6 months (range, 0.9 to 39.8 months), 117 cases were alive. Thirty-three cases in the standard group and 35 in the intensified group died. Comparing standard versus intensified regimens, the causes of death included leukemia relapse (n = 18 vs 14), GVHD (n = 7 vs 8), infections (n = 5 vs 7), RRT (n = 2 vs 0), EBV-associated diseases (n = 0 vs 5) and pulmonary haemorrhage (n = 1 vs 1). The 3-year OS and DFS in the standard group were 62.2% ± 5.8% and 60.6% ± 5.6%, compared with 51.6% ± 6.2% and 51.1% ± 5.9% in the intensified group, respectively (*P* = 0.029, *P* = 0.063, log-rank test). The 3-year cumulative incidence of relapse and nonrelapse TRM were 28.1% ± 5.7% and 18.1% ± 4.8% in the standard group, compared with 38.2% ± 8.9% and 27.8% ± 5.2% in the intensified group, respectively (*P* = 0.209, *P* = 0.030, log-rank test).

## Discussion

In allo-HSCT, the relapse of the underlying disease is the main factor that affects survival. The intensity of conditioning regimen has been shown to directly affect the relapse and survival [30,31]. Some studies suggested that intensified conditioning could reduce tumor relapse, but it might simutaneously increase TRM including infection-related mortality [10-12]. In addition to the anti-tumor effect of conditioning regimens, the therapeutic efficacy of allo-HSCT also relies on the graft-versus-tumor (GVT) effect [1,32]. In this study, based on the results of our previous studies [13], we introduced the regimen of intensified conditioning, early tapering of prophylactic immunosuppressants followed by DLI for inducing GVT effect for patients with high-risk and refractory hematological malignancies, with 3-year OS and DFS of 51.6% ±6.2% and 51.1% ± 5.9%. The results once again proven that intensified conditioning followed by inducing GVT effect was effective for patients with high-risk and refractory hematological malignancies.

Infections are another leading cause of death after allo-HSCT. Some studies reported that the incidence of infections and the infection-related mortality might reach up to 77% and 20% after allo-HSCT, respectively [3,33,34]. Recently, with wide applications of antibacterial and antifungal drugs in the prophylaxis and therapy of infections, the incidence and mortality of bacterial and fungal infections post-transplantation decrease markedly. However, due to the absence of effective preventive and therapeutic drugs for most viruses, the incidence and mortality of viral infections increase relatively, especially in the early period after transplantation. Some studies suggested that intensified conditioning was accompanied by an increasing incidence and mortality of early-stage infections, due to aggravated tissue and organ damage as well as the delay of immune reconstitution after HSCT [10-12]. In this study, we prospectively compared the effects of standard and intensified conditioning on infections, especially EBV and CMV infections. Our data further confirmed our previous results that intensified conditioning did not increase the incidence and mortality of bacterial and fungal infections early post-transplantation [13]. Meanwhile, our data showed that intensified conditioning might increase the incidence of EBV viremia and EBV-associated diseases as well as the mortality of EBV-associated diseases, but it did not affect the incidence of CMV viremia and CMV-associated diseases as well as the mortality of CMV-associated diseases. The differences might be associated with the fact that there was optimal strategy for prevention and treatment of CMV infections, but lack of effective methods to prevent and treat EBV infections.

Although EBV and CMV infections are the most common opportunistic viral infections and closely related to the immune function, the risk factors for both infections are different in recipients of allo-HSCT. Recognized main risk factors for EBV infections include T-cell depletion, use of ATG or anti-CD3 monoclonal antibody, HLA mismatch, unrelated donor and so on [35-38]. Important risk factors for CMV infections are associated with the serological status of donor and recipient, aGVHD, T-cell depletion and use of ATG [18,23]. In this study, we analyzed the risk factors for EBV and CMV infections. Univariate analysis revealed that HLA mismatch, unrelated donor, use of ATG, advanced disease status and aGVHDII-IV were associated with EBV infections; use of ATG, aGVHDII-IV, HLA mismatch, early CsA withdrawal and DLI were associated with CMV infections. Upon multivariate analysis, use of ATG was found to be the risk factor for EBV viremia and EBV-associated diseases; HLA mismatch and early CsA withdrawal were the risk factors for CMV viremia; aGVHDII-IV was the only risk factor for CMV-associated diseases. These results were consistent with current studies [18,23,36,38], except the finding that CMV infections was associated with early CsA withdrawal and DLI. The reasonable explanation for this finding was that early CsA withdrawal or DLI could increase the incidence of GVHD, and GVHD was the risk factor for CMV infections. Interestingly, univariate and multivariate analysis both revealed that intensified conditioning was the risk factor for EBV viremia and EBV-associated diseases. The mechanisms that intensified conditioning increased EBV infections might be associated with the effects of intensified conditioning on early immune reconstitution. Therefore, we analyzed the immune reconstitution of recipients early post-transplantation and found that the percentage of CD4^+^ T cells and ratio of CD4^+^/CD8^+^ T cells at 3 months post-transplantation were significantly lower in the intensified group.

## Conclusions

Intensified conditioning represents a promising approach for high-risk hematological malignancies, although it affects early immune reconstitution of recipients and increases the incidence and mortality of EBV infections.

## Competing interests

The authors declare that they have no competing interests.

## Authors’ contributions

LX performed investigations, analyzed data and wrote the paper; FH, ZPF, HSZ and XZ analyzed data; GPY, YZ, CL and JS performed investigations; QFL designed the study and wrote the paper. All authors read and approved the final manuscript.
